# Influenza A Gradual and Epochal Evolution: Insights from Simple Models

**DOI:** 10.1371/journal.pone.0007426

**Published:** 2009-10-20

**Authors:** Sébastien Ballesteros, Elisabeta Vergu, Bernard Cazelles

**Affiliations:** 1 UMR 7625 (UPMC, ENS, AgroParisTech, CNRS), École Normale Supérieure, Unit of Eco-Evolutionary Mathematics, Paris, France; 2 INRA, UR341 Mathématiques et Informatique Appliquées, Jouy en Josas, France; 3 UMMISCO UMI 209 IRD-UPMC, Bondy, France; University of California, Berkeley, United States of America

## Abstract

The recurrence of influenza A epidemics has originally been explained by a “continuous antigenic drift” scenario. Recently, it has been shown that if genetic drift is gradual, the evolution of influenza A main antigen, the haemagglutinin, is punctuated. As a consequence, it has been suggested that influenza A dynamics at the population level should be approximated by a serial 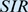 model. Here, simple models are used to test whether a serial 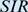 model requires gradual antigenic drift within groups of strains with the same antigenic properties (antigenic clusters). We compare the effect of status based and history based frameworks and the influence of reduced susceptibility and infectivity assumptions on the transient dynamics of antigenic clusters. Our results reveal that the replacement of a resident antigenic cluster by a mutant cluster, as observed in data, is reproduced only by the status based model integrating the reduced infectivity assumption. This combination of assumptions is useful to overcome the otherwise extremely high model dimensionality of models incorporating many strains, but relies on a biological hypothesis not obviously satisfied. Our findings finally suggest the dynamical importance of gradual antigenic drift even in the presence of punctuated immune escape. A more regular renewal of susceptible pool than the one implemented in a serial 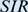 model should be part of a minimal theory for influenza at the population level.

## Introduction

Currently, two subtypes of influenza type A virus (H3N2 and H1N1) cocirculate in human populations along with the influenza type B virus. In temperate zones and during inter-pandemic periods, their dynamics lead to annual epidemics of variable amplitude caused by alternating types and subtypes [1]. Worldwide, these annual epidemics result in about three to five million cases of severe illness, and about 250 000 to 500 000 deaths [2].

The recurrence of influenza A epidemics is still not thoroughly understood despite a large amount of empirical and theoretical investigations. It has originally been explained by the evolution of the main surface glycoproteins of the virus (mainly haemagglutinin, HA, but also Neuraminidase, NA) inducing possible “reinfection” of previously infected hosts. This “continuous antigenic drift” scenario [3] where viruses continuously escape immunity as mutations accumulate has recently been challenged by new sequences data and theoretical developments.

From the theoretical side, multi-strains models tracking the infection history of the hosts have been difficult to use due to the exponential growth of state variables as the number of strains increases [4]. Nevertheless, by using a status based approach combined with the assumption that a previous infection reduces infectivity and that co-infections are allowed, [5] have produced a model where the number of state variables grows linearly with the number of strains. It has thus been possible to study how immunologically cross-reactive strains sequentially invade a partially susceptible population. The results of [5] model, using a linear antigenic space, have shown a self-organisation of the strains into antigenic clusters. This organisation results in a punctuated antigenic evolution based on a continuous genetic change, challenging the idea of a gradual antigenic drift.

From the observational and experimental side, [6] have mapped the antigenic and genetic evolution of influenza virus from real data using statistical techniques. They have confirmed the theoretical results of [5], with antigenic clusters emerging and replacing each other every 2 to 8 years.

Other theoretical works have enabled to relax the hypothesis of a linear antigenic space [7], [8]. Such a gain in realism has resulted in an intuitive explosion of strains diversity due to a positive feedback. As the antigenic diversity of co-circulating strains increases, the production of further variants is also increased. The key theoretical question has thus been to explain how the strain diversity could be restricted to be compatible with the phylogenetic tree of the glycoprotein HA of the subtype H3N2 [9]. Ferguson et al. (2003) (see also [10], [11]) have included in their model a strain transcendent temporary immunity (previously suggested by [12]), along with some sources of variability [13]. This approach allows simulating realistic viral evolution at the sequence level. Nevertheless, it remains difficult to prove conclusively the physiological support of this non permanent immunity through appropriate experiments.

Recently, [14] have been able to reproduce the dynamics of influenza HA genetic diversity within a high dimensional antigenic space without invoking the temporary cross-immunity. [14] model focuses on antigenic clusters resulting from a degenerated genotype to phenotype map. The authors have considered that the evolution of the main antigen of influenza A has two principal characteristics: first, it consists of long periods of stasis where antigenic clusters globally do not change their antigenic properties but evolve through neutral or almost neutral mutations; second, these periods are punctuated by bursts of positive selection which precipitate antigenic cluster transitions due to rare escape mutations. The occurrence of new antigenic clusters results in selective sweeps that restrict strains diversity. [14] model have shown that weak within cluster selection and the selective sweeps that accompany antigenic clusters transition are sufficient to recover most of HA interpandemic evolutionary dynamics, a finding confirmed by genetic data analyses [15], [16]. [14] results suggest a new starting point for the investigation of influenza dynamics at the population level.

Here we are interested in the consequences of [14] results at the population level. Contrary to the classical 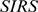 model of [3], which resorts to a gradual antigenic drift, [14] results suggest to focus on a serial 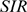 model with discrete 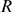 to 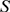 transitions provoked by punctuated evolution (rare immune-escape mutants with strong antigenic effects). We are interested in contrasting the serial SIR paradigm and the classical SIRS model of [3]. In particular, we seek to determine whether a serial 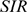 model would require gradual antigenic drift within clusters. As revealed by [14] study, gradual antigenic drift favours antigenic cluster change by facilitating the antigenic space exploration and also increases susceptible renewal. Our approach mainly neglects the epidemiological impact of gradual antigenic drift to disentangle the complex causal links induced by the interactions between births and deaths processes, gradual antigenic drift, clusters change, external virus reintroductions and specific modelling assumptions. Our objective is to use simple and tractable models to determine to what extent a serial 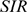 model per se, *i.e* neglecting gradual antigenic drift can constitute a minimal model for influenza A dynamics at the population level.

Our analysis mainly focuses on transient dynamics that appear of first importance for selective sweeps and antigenic clusters replacement. To our knowledge, contrary to what has been done for the stationary dynamics (see [17]), no study has focused on the consequences of these modelling assumptions on the *transient* dynamics.

From the methodological side, we start by clarifying the effects of classical modelling assumptions of multi-strains SIR models on the invasion and persistence of a new antigenic cluster. History and status based two-strain models including reduced infectivity and susceptibility assumptions are considered (section Methods). Significance and choice of biologically relevant numerical values for model parameters are then discussed. The deterministic framework is first explored (sections Results). Then, both stochasticity and external reintroduction of viruses are added in order to test the robustness of the obtained transient dynamics. Finally we discuss the biological limitations of the only model able to reproduce observed antigenic cluster replacement dynamics and, more generally, the ingredients of a minimal theory for influenza A. Our findings globally suggest the impact of the modelling assumptions on the outcome of the invasion of a new antigenic cluster. They also stress the dynamical importance of gradual antigenic drift in a minimal theory for influenza at the population level even in the presence of punctuated immune escape.

## Methods

In order to explore the behaviour of the serial 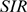 model as a minimal theory for influenza, we consider an adaptive dynamics framework [18]. The *resident* population is an antigenic cluster of influenza strains at endemic equilibrium, illustrating the long period of stasis described by [16]. The immune escape mutation (as a consequence of a true mutation or a re-assortment [19]) generates a new antigenic cluster called the *mutant*. We are interested in the outcome of the invasion of the resident viral population by the mutant. This framework illustrates the burst of positive selection proposed by [16].

### Different assumptions for the modelling of partial cross-immunity for co-circulating antigenic clusters; deterministic framework

We study the outcome of immune escape mutations, by using two-strain dynamical models applied to the resident and the mutant antigenic clusters defined here above.

Two main modelling approaches have been used to study immunologically cross-reactive strains: *(i) history based (HB) models* [4] and *(ii) status based (SB) models* [20]. As stressed by [20] and [21], in a *HB* model, all hosts previously infected by a strain 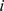 become partially immune to a second strain 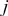. In *SB* model, when a given host gets infected by a strain 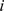, the within-host immunological dynamics takes place and “immediately” generates the immunological status (immunised or not) towards strain 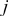 [20].

Partial cross immunity can be modelled using two extreme hypotheses: *(i) reduced infectivity (RI)* or *(ii) reduced susceptibility (RS)*. 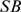 models with 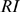 assumption exhibit the attractive mathematical property of dimensional reduction without loss of information, containing twice more equations that strains. The tractability of this kind of models has been exploited in previous works [5], [14], [22].

To clarify the effect of these various assumptions, we provide a comparison of both 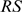 and 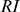 cases in both 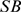 and 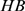 models.

#### Status based model with reduced susceptibility *(SBRS)* and co-infections

We introduce the following notations: 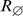 is the proportion of hosts with no acquired immunity, 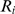 is the proportion of hosts who have acquired immunity to cluster 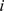 and 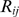 is the proportion of hosts who have acquired immunity to clusters 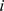 and 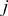. Note that we include currently infected hosts (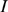) into the 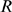 state variables. Partial cross-immunity is modelled by 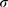, which represents the probability of being immunised against cluster 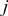 when infected by cluster 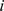.

Using these notations and considering that co-infections are possible during the infectious period and that infections with one antigenic cluster reduce susceptibility to the other, we can derive (see for instance [21] or [20] ) equation (1):
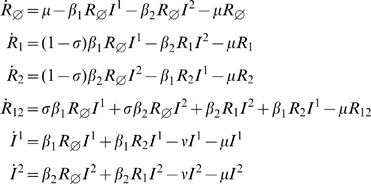
(1)


Parameter interpretation and values are given in Table 1.

**Table 1:** Parameter values.

Parameters	Theoretical	Empirical
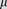 (birth and death rate)	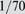 years 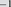 [28]	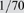 years 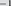 [28]
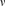 (recovery rate)	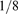 days 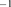 [14]	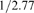 days 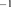 [30]
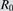 ( 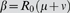 )	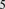 [14]	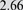 [30]

#### Status based model with reduced infectivity *(SBRI)* and co-infections

In the case where infection by one antigenic cluster reduces the infectivity of a subsequent infection by the other cluster, using the same notation as in (1) and still allowing coinfections during the infectious period, we obtain:
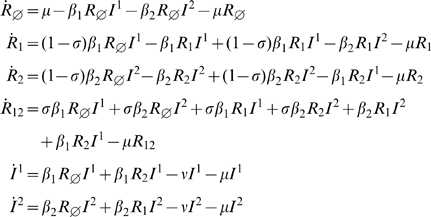
(2)


A precise derivation of (2) can be found in the appendix of [21]. This model can be further reduced to four equations by defining 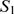 and 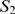 as 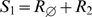 and 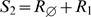 respecitively. This leads to a two-strain version of the model of [5].

#### History based model

In this framework, notations are changed to follow the infection history of the hosts. Hosts can be susceptible to both clusters (proportion 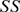), susceptible (or resistant) to one cluster and infectious with the other one (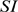 and 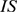, or 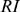 and 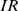 respectively), infectious with (or resistent to) both clusters (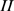 or 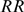 respectively) or susceptible to one cluster and resistant to the other one (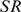 and 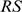 respectively).

When first immunised by one cluster, hosts can be less infectious when infected by the second cluster: the infectivity is modulated by the parameter 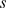 and the model is called the history based model with reduced infectivity (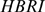). Hosts can also have a reduced susceptibility towards the second cluster controlled by parameter 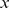. The model is called the history based model with reduced susceptibility (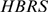).

This gives rise to the following equations (3), with 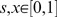.
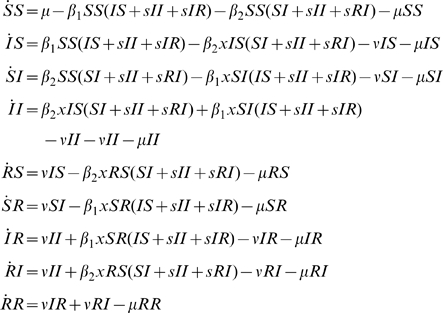
(3)


As noted by [23], in the case of the *RS* assumption we can reduce the dimension of the system by introducing the following state variables: 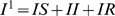; 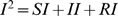; 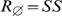; 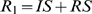 and 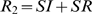.

Another assumption was used by [24]. In [24] model, cross-protection does not affect susceptibility but reduces transmissibility by a factor 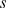. Instead of reducing the infectivity of the hosts as for the previous 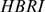 model (equation 3), [24] model assumes that infection by a cross-reactive cluster of partially protected hosts results in a partition of the infected hosts into a proportion 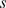 of infectious hosts and a proportion 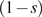 of non infectious hosts that nevertheless become immunised to the infecting cluster. This assumptions lead to equation 4.
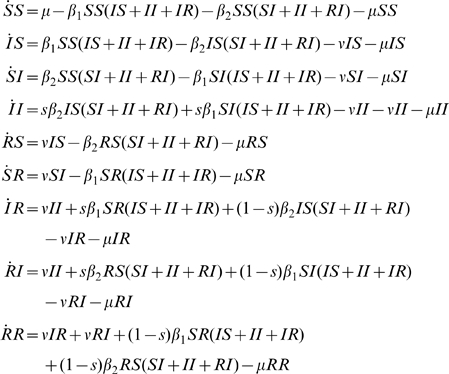
(4)


**Figure 1:**
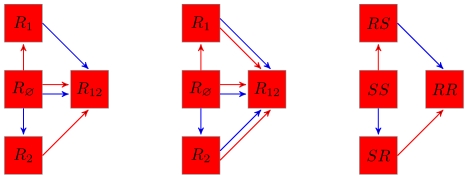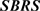 (left), 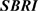 (middle) and 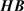 (both 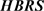 and 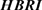) (right) two antigenic-clusters models. Red (blue) arrows represent infection by antigenic cluster 1 (2). Only the 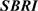 model (middle) is subject to cross-immune boosting (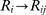 following reinfection by strains of cluster 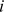).

As originally proposed by [24] the dimension of equation 4 model can be reduced by introducing: 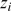 the proportion of hosts infectious or immunised by cluster 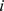 (*e.g* 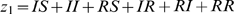), 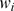 the proportion of hosts infectious or immunised by cross-reactive cluster with cluster 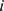 including cluster 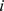 itself (*e.g* 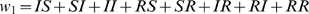) and 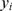 the hosts infectious by cluster 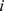 (*e.g* 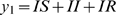). In case were the degree of protection against new infections is the same for all related strains, [24] model contains only three times more equation than strains. However, generalisation to several levels of cross-protection greatly increases the dimensionality [11], [25]. As the model in equation 4 and the 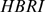 model of equation 3 lead to similar results, we will only consider the latter one depicted by equation 3. Our analyses will thus concern four models: 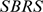, 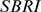, 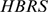 and 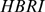 all summarised in figure 1.

### Stochastic models

We implemented stochastic versions of each of these four models (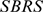, 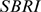, 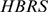 and 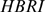) using Gillespie event-driven algorithm [26] and the MT19937 random number generator of Makoto Matsumoto and Takuji Nishimura provided by the C library GSL [27]. For instance, for the 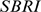 model, the differential equation system (2) can be translated into the reaction scheme described in Supporting Information S1.

### Parameter values

Two sets of parameters were used here. One consists of parameters mainly used in theoretical papers (*e.g.* [7], [14], [28]) and the other consists of more direct estimates of parameters from household studies (*e.g.* [29]–[31]). They are all defined in Table 1. For comparison purpose, we have retained the parameters values of [14] and have provided a sensitivity analysis using the other set of parameters (results not shown). Parameters 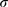 and 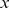 (or 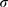 and 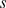) can be related by 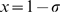 (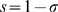 respectively). For the sake of simplicity, we refer to 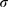 only and express 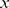 and 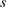 with respect to 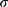.

The choice of appropriate values for 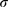 was motivated by the significance of the process captured by the model. We are mainly interested in antigenic evolution occurring during epidemic influenza (whether it is *punctuated* or *gradual*). As we work at the phenotype level, our framework can also be used to study pandemic influenza and *antigenic shift* (appearance of new influenza subtypes within humans). The distinction between these three processes (gradual/punctuated antigenic drift and antigenic shift) is only based on the value of 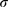 (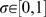) taken as a bifurcation parameters. Low 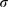 values (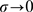) are related to *antigenic shift* and 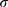 values close to 1 correspond to *antigenic drift*, either gradual or punctuated. In order to separate punctuated from gradual antigenic drift, we use the scale given in [14] and consider that 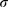 values under 0.93 are relevant for punctuated immune escape (typically 0.8), whereas higher values, closer to 1, are more appropriate for gradual antigenic drift. Note that comparable values were used in previous studies focusing on gradual antigenic drift [5], [7]

**Figure 2:**
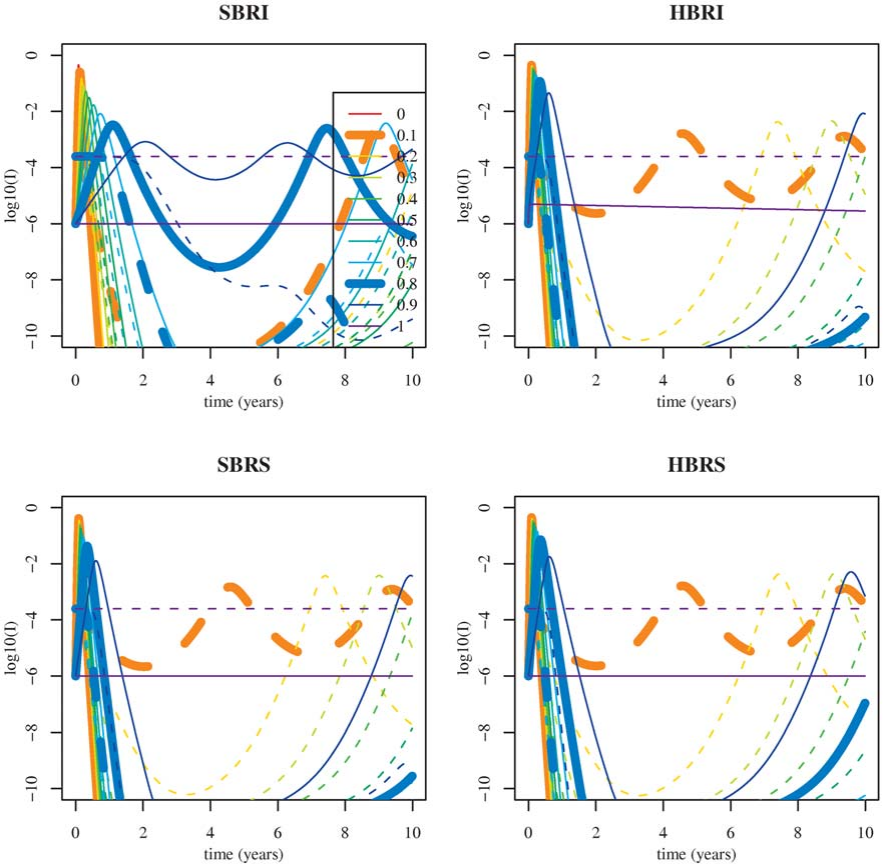
Transient invasion dynamics for the four two-cluster models studied. The decimal logarithm of the proportion of infectious hosts for the mutant antigenic cluster (plain lines) and for the resident cluster (dashed lines) is represented as a function of 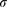. Colours correspond to different partial cross-immunity (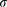) values: from 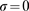 (antigenic shift, no cross-immunity) to 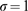 (antigenic drift, full cross-immunity). Parameter values are given in Table 1 (theoretical set). Initial conditions are: 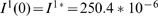, 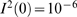.

Defining the population size (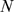) is of tremendous importance when using stochastic models [32]–[36]. As we are mainly interested in replacement dynamics, we need to define a population size where the resident cluster can persist when alone in order to avoid confusion between different causes of replacement. According to our simulations (Supporting Information S1), we choose a population size of 10 million of individuals to ensure that resident extinctions are not due to endemic fadeout during the timescale considered (10 years, figure 2). This value is also used in the deterministic framework to fix a threshold (equal to 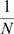) below which we consider that extinctions occur. Note that this Critical Community Size (CCS) [37] does not guarantee that the resident strain could have invaded the population and persist [38].

## Results

### Invasion condition of the mutant cluster

We start our analysis by examining the dynamical impact of the four modelling assumptions (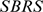, 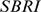, 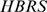 and 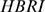) corresponding respectively to equations (1), (2) and (3)) thorough calculation of invasion conditions of the mutant cluster (labelled cluster 2) within the environment corresponding to the equilibrium of the resident cluster (labelled cluster 1).

For the 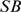 models, in both 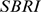 and 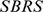 versions, the invasion condition can be deduced from:
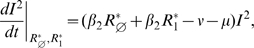
(5)where 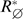 and 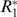 are equilibrium values of 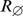 and 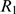 when only cluster 1 is present. In both 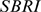 and 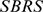 models, 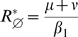. For 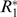, in the 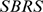 model 
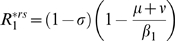
 and 
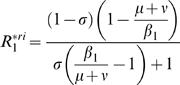
 in the 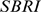 model, that is 
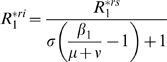
. If parameters are equal for the two antigenic clusters (that is 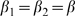), equation (5) becomes:
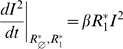
The mutant can invade (*i.e.* 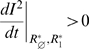) as long as 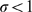 provided that 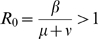. The invasion fitness of the 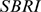 model equals the one of the 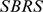 model divided by 
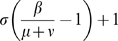
. Depending on 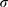, the initial speed of invasion with the *RI* assumption can be greatly decreased. *RS* and *RI* assumptions are not without effects on the transient dynamics of antigenic clusters invasion in SB models.

In the *HB* framework, the previous approach is not feasible for the 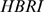 model. The basic reproduction ratio 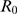 is calculated in this case as the dominant eigenvalue of the linear next generation operator [39]. In both 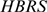 and 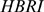 models the dominant eigenvalue is:
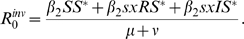
As for the *SB* cases, 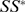, 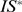 and 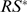 are equilibrium values of 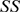, 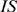 and 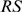 when only cluster 1 is present and are equal to 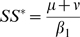, 
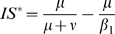
 and 
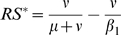
. If 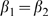, the invasion is possible (*i.e.* 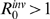) as long as 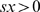 provided 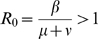. Contrary to the *SB* framework, in a two-cluster *HB* model, invasion fitness is the same in both *RS* and *RI* cases.

Table 2 provides a comparison of the 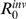 for the four models considered and reveals that the 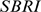 model differs from the three others which possess the same 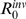. The 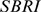 model assumption appears to reduce the initial speed of invasion of the mutant cluster by a factor 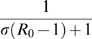.

**Table 2:** Invasion 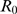.

model	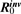
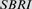	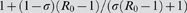
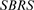	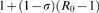
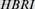	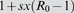
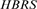	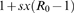

Comparison between the four two-cluster models (equations (1), (2) and (3)) in terms of the basic reproduction ratio (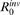) of the mutant cluster invading a resident population at endemic equilibrium.

### Invasion and extinction

#### Deterministic framework

**Figure 3:**
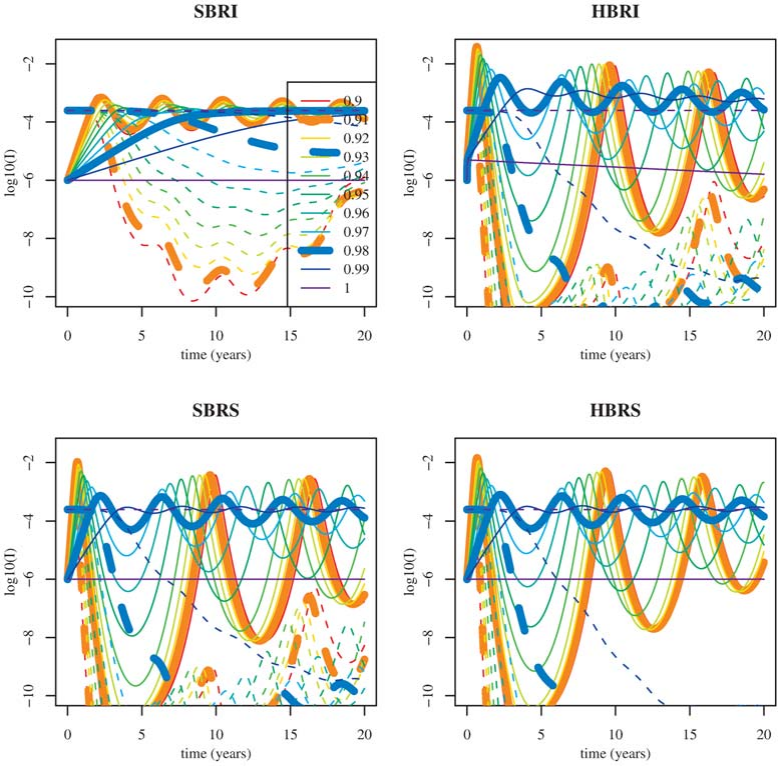
Detail of figure 2. Partial cross-immunity (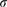) values more relevant for gradual antigenic drift (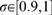).

**Figure 4:**
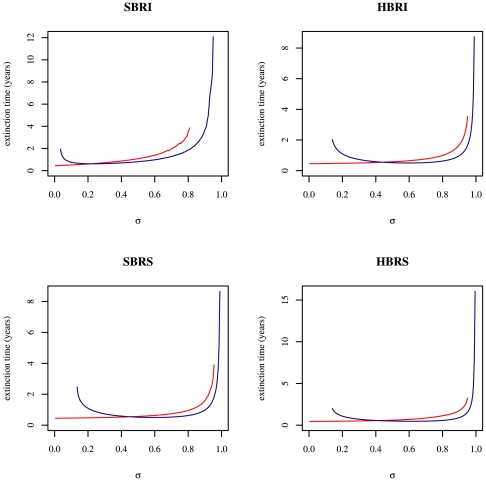
Extinction times of the resident antigenic cluster (blue) and of the mutant cluster (red) for the four two-cluster models studied. Parameter values are given in Table 1 (theoretical set). Initial conditions are: 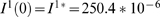, 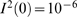.

Figures 2, 3 and Supporting Information S1, illustrate a comparison of the effect of 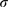 on the invasion dynamics of a new cluster (the resident being at endemic equilibrium) for the four two-cluster models studied with parameters set at theoretical values (Table 1). Figure 4 summarises the results of the transient dynamics of the mutant invasion in terms of clusters replacement considering a deterministic threshold of 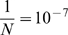 for extinction as determined by simulations summarized in Supporting Information S1.

For 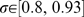 (corresponding to [14] scale of rare immune escape mutations) in figures 2 and 4 antigenic cluster replacements are possible only for the 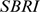 model. The three other models exhibit extinction of both antigenic clusters. For 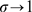 (corresponding to gradual antigenic drift) in figures 3 and 4 the 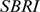 model results in coexistence of both clusters contrary to the three other models which predict the replacement. For 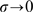 (antigenic shifts) in figure 2 and 4 the resident influenza subtype is not sufficiently affected by the mutant subtype to go extinct and it survives while the mutant disappears after generating an outbreak. Note that smaller values of cross-immunity are sufficient for the 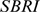 model to drive the resident to extinction (figures 2 and 4).

In all cases, a proper rescaling of the 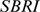 model with lower 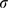 values as suggested in Table 2 is needed to render it comparable to the three others models.

#### Stochastic framework

**Figure 5:**
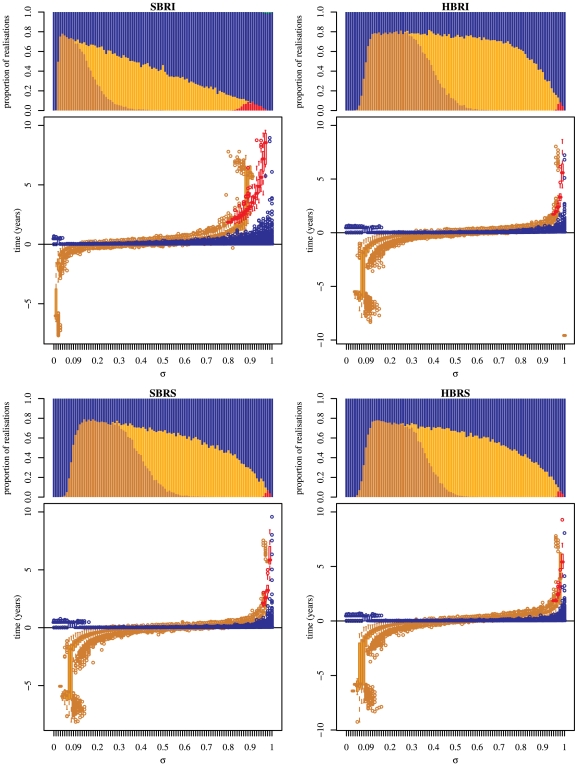
Outcomes of the transient invasion dynamics based on 1000 realisations of the four two-cluster stochastic models. For each panel, top graphs represent the proportion of realisations where, after 10 years: both antigenic clusters go extinct, but the mutant goes extinct first (brown); both antigenic clusters go extinct, but the resident goes extinct first (aborted replacement, orange); the resident cluster only goes extinct (successful replacement, red); the mutant cluster only goes extinct (blue); no cluster goes extinct (coexistence, green). For each panel, bottom box plots represent: extinction times of the mutant cluster when only this cluster goes extinct (blue); extinction times of the resident cluster when only this cluster goes extinct (red); the differences between extinction times of the mutant cluster and the resident when both clusters go extinct (brown and orange). Initial conditions: one infected individual with the mutant antigenic cluster is introduced in a population where the resident cluster is at the deterministic endemic equilibrium. The remaining initial conditions are those corresponding to the endemic equilibrium of the deterministic model and parameter values are given in Table 1 (theoretical set).

Simulated trajectories corroborate the trends provided by deterministic models, especially the particularity of the 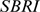 model (figures 5, and Supporting Information S1).

The replacement of antigenic clusters following rare mutations with strong antigenic effect appears to be realistic only in the case of the 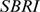 model (figure 5, red bars and Supporting Information S1) for which a set of 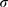 values consistent with punctuated immune escape variability exists. For these 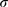 values (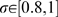), a trade-off exists between invasion ability (that is risks of initial extinction) and risk of epidemic fade-outs (as described for the evolution of the recovery rate by [40]). Figure 5 shows that the proportion of initial extinctions, previous to an epidemic caused by the mutant, decreases as long as the degree of immune escape (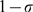) increases (blue colour in panel 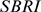 of figure 5). At the same time, the proportion of epidemic fade-outs after replacement increases (orange colour in panel 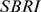 of figure 5). Moreover, these results are consistent with formulas given in Table 2, since the probability of initial extinctions of the mutant cluster is given by 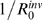 [39]. For the 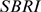 model, this probability increases linearly with 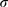 (figure 5 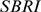, blue bars) whereas for the three other models (panel 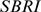, 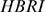 and 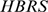 of figure 5, blue bars), it remains uniformly lower and increases as 
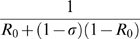
 with 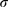.

The time necessary to drive the resident cluster to extinction is also a decreasing function of the immune escape intensity (red boxplot in panel 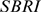 of figure 5). For 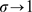, transient coexistence (5 years) of both antigenic clusters is expected before definitive replacement.

Taken together, the previous results reveal that: *(i)* antigenic clusters replacement within a serial 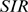 model is possible only in the case of a 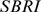 model; *(ii)* antigenic shift results in the extinction of both subtypes (brown colour figure 5, trajectories in Supporting Information S1) or of the mutant only (blue colour figure 5).

### External re-introductions

#### Modelling re-introduction

In the real world, populations are opened to migration and extinct clusters can be re-introduced. To complement our results we need to evaluate the timescales of re-invasion. In particular, we focus on: *(i)* the robustness of the replacement (i.e. is the resident able to re-establish in the population due to spatial effects of re-introduction?); *(ii)* which cluster re-invades first when both are extinct quasi simultaneously.

Except for initial extinctions, the observed extinctions are mostly due to deterministic forces of susceptibles depletion and not to random fluctuations of trajectories evolving close to one individual (low variances in the box plots of figure 5). Incidentally, the opportunity of a second epidemic after an epidemic fadeout for the mutant cluster or, the opportunity of re-invasion of the resident cluster after having been extinct due to the invasion of the mutant cluster are mostly governed by the deterministic dynamics of susceptibles renewal [41].

We will thus use deterministic models to compute the average time necessary before a recurrent epidemic. A simple way to do this is to consider a constant amount of infectious individuals entering the population studied. Classically (*e.g.* [42], [43]) the following scheme has been used:
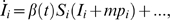
where 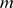 is the number of infected individuals imported from outside (generally 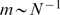) and 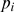 is the proportion of these immigrating hosts infected with strain 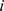. Note that we do not consider infecteds from outer regions in the bookkeeping of 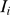.

From Supporting Information S1 we can see that the overall pattern of transient dynamics is not affected by the modelling of external re-introductions.

### Re-invasion time-scales

**Figure 6:**
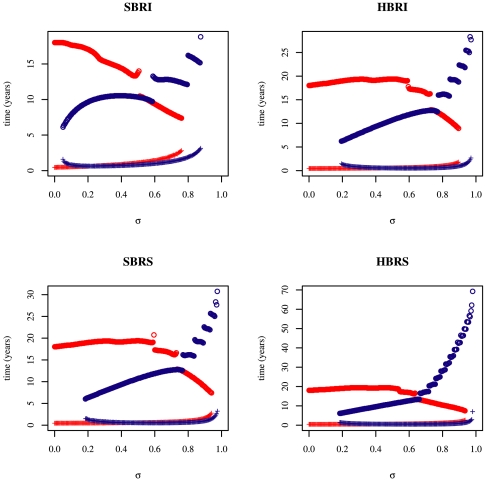
Extinction and re-invasion times for the four two-cluster models in the presence of external reintroductions of infectious hosts. (+) represent times when a deterministic threshold (equal to 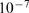) for extinction is crossed by the trajectories for the resident cluster (blue) and the mutant (red); (o) correspond to times of the first peak after extinction for the resident cluster (blue) and times of the second peak of the mutant cluster (red). Parameter values are given in Table 1 (theoretical set), 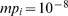. Initial conditions are: 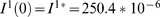, 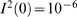.

Figure 6 reveals that for 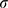 values relevant for punctuated antigenic drift (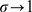), successful replacements are robust to the re-introduction of the resident antigenic cluster (*i.e* the re-introduction of the resident cluster does not lead to an epidemic). In the case of replacements where both clusters go extinct (the resident being extinct before the mutant) the mutant cluster re-invades first. This underlines the fact that we face a replacement. The time until the next epidemic is nevertheless unrealistically high (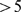 years) to be consistent with observed patterns of influenza yearly recurrence in the absence of antigenic cluster changes.

For antigenic shifts (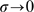), when both clusters go extinct, timescales for a recurrent epidemic are also too long to be relevant (re-invasion time 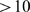 years, figure 6). In the case where the invader is able to drive the resident to extinction (that is for the 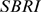 model), replacements are not robust to external re-introduction. The former resident re-appears more than 10 years before the invader.

## Discussion

Punctuated antigenic evolution is being recognised as an important mechanism of immune escape in various RNA viruses, but its detection remains difficult and somewhat uncertain [44]. In this paper we have focused on exploring to what extent the complex processes shaping influenza dynamics can be approximated by a minimal serial 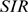 system, emphasising rare mutations with strong antigenic effects. According to our results (figure 2, 3), punctuated immune escape results in a high depletion of susceptibles in 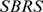, 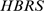 and 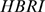 models. As a consequence, recurrent epidemics during consecutive years are rendered impossible even with reintroductions. However, data clearly suggest that several recurrent epidemics of the same new mutant cluster can follow the replacement of the resident cluster by the new one. For instance, following its invasion, Beijing/1993 (BE93) cluster has provoked epidemics of 1992–1993, 1993–1994, 1994–1995 and 1995–1996 seasons in New York state before being replaced by Wuhan/1995 (WU95)-like viruses [45]. Such dynamics can only be reproduced by the *SBRI* model because it produces comparatively slower invasion dynamics and fewer susceptible depletion. A minimal serial *SIR* theory is thus supported only within the 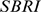 framework.

In the following, we review the processes that makes the 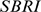 model different from *HB* or *SB* models with *RI* assumption. We then provide elements pointing out that these processes direct towards a biologically problematic description of cross-immunity. Finally, we provide arguments supporting the idea already evoked by [14] that a sequential 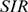 model requires within antigenic cluster gradual antigenic drift and that this process should be part of a minimal theory for influenza dynamics at the population level.

### Is the 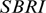 model particularly appropriate?

One of the important aspects of influenza dynamics is the cross-immunity represented here by the parameter 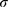 which measures the antigenic distance between two strains, regardless of the modelling framework. Here, the range of variation of 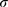 was the same for the four models and was chosen according to [14]. This allowed direct comparison between the four models.

Our results reveal that the similar dynamics are generated for significantly higher values of 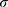 in the case of the 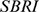 model than for the other three models (Table 2). This difference in behaviour is due to the fact that in the 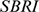 model, individuals that have been infected with cluster 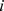 can be reinfected by the same cluster. These reinfected hosts will not be infectious (because of the *RI* assumption) but may enhance their immunity to cluster 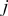 (figure 1, middle). In equation (2) repeated infections corresponds to the terms 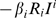. 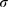 percent of these hosts acquire immunity to strain 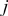, progressing to the 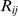 status whereas the remaining 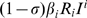 hosts keep the 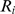 status. As noted by [21], such cross-immune enhancement is impossible in the 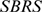 model because by construction of this latter model 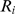 hosts are no more susceptible to cluster 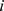 and cannot be reinfected.

In the context of influenza, cross-immune enhancement as provided by the 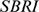 model appears to contradict established theory for immunodominance, cross-reactivity and interference (see [46] for a review). For sequential infections, a key question is to determine whether a new infecting strain is sufficiently different from a previously encountered strain to consider that a new primary response would be mounted by the immune system instead of a secondary response. In our model, we considered that independent primary responses were mounted for the different antigenic clusters. Strains belonging to cluster 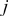, were supposed sufficiently different from strains belonging to cluster 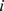 not to interact with memory cells supporting immunity toward strains of cluster 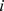. The reinfection then results in the production of 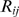 hosts in both *SBRS* and *SBRI* models. For the case of reinfection of 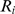 hosts with closely related strains belonging to cluster 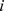 (possible only for the *SBRI* model), one can reasonably assumes that such strains are sufficiently closed to interact with the memory cells (otherwise they would belong to cluster 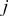). In this case, according to [46], we can expect a sequential effect called original antigenic sin, well known for influenza [47]–[49]. Within original antigenic sin, a strong response against a previously recognised epitope represses the response against the changed epitope. As the *SBRI* model assumes a strong and immediate response toward the previous epitope (hosts reinfected with a virus from an identical cluster are no longer infectious), the rapid response from memory cells may keep viral load below the threshold required to stimulate naive B or T cells (other processes are also possible [49]).

Given these mechanisms, the cross-immune enhancement provided by the 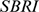 model should be considered as an overestimation bias of immunity and proper rescaling of 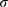 should be done before using the 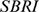 model in the context of influenza.

### Toward a minimal theory for influenza

Except for the biologically problematic 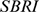 model, our results stress that the occurrence of new antigenic clusters resulting from immune escape mutations rapidly induces important depletion of susceptibles. This depletion results in an extinction of the invading antigenic cluster and this phenomenon is robust to reintroductions (figure 2, 3 and 6). Thereafter, we propose processes that can favour the replacement of the resident by the mutant as observed in data.

#### Gradual antigenic drift within antigenic clusters

[28], have considered a model that incorporates gradual antigenic drift within antigenic clusters. They have assumed that within cluster evolution results in a diversity of strains that renders immunity to an antigenic cluster only partial. Partial immunity has been modelled by a 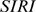 model [50], allowing reinfection at a slower rate. [28] have shown that reinfections define a reinfection threshold [50], [51] that plays a central role in determining the outcome of the invasion by a new antigenic cluster. Reinfection determined by gradual antigenic drift therefore appears to be central for successful antigenic cluster replacement as observed in data. Contrary to [28] claims that no antigenic cluster replacement can occur within 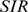 models, we have shown that this could be the case with 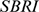 models. However, since the 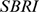 model is biologically problematic, it still remains to be tested whether 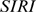 or 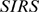 models would best describe drifting antigenic cluster.

**Figure 7:**
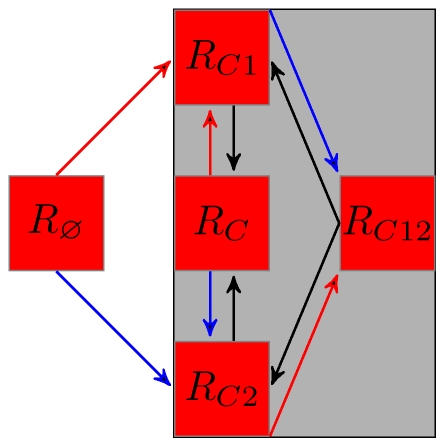
An history based model for drifting co-circulating cross-reactive antigenic clusters. The viruses are supposed to contain two antigens: a conserved antigen, shared by strains of the resident and the mutant antigenic cluster and a specific antigen, specifying each cluster. Naive hosts acquire immunity to both conserved and specific part (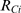) resulting in full protection toward strains of cluster 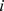. Within cluster antigenic drift affects only the specific antigen resulting in 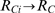 transitions at a rate governed by parameter 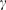. The shared conserved antigen confers partial protection reducing the probability of reinfection by a factor 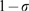. Red (blue) arrows represent infection by cluster 1 (2). Black arrows represent within cluster antigenic evolution. A full description of the assumptions leading to this model is provided in section 4 of Supporting Information S1. These hypotheses also enable to recover the model of [28] and therefore render the two frameworks (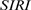 and 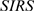 within cluster antigenic drift description) readily comparable.

**Figure 8:**
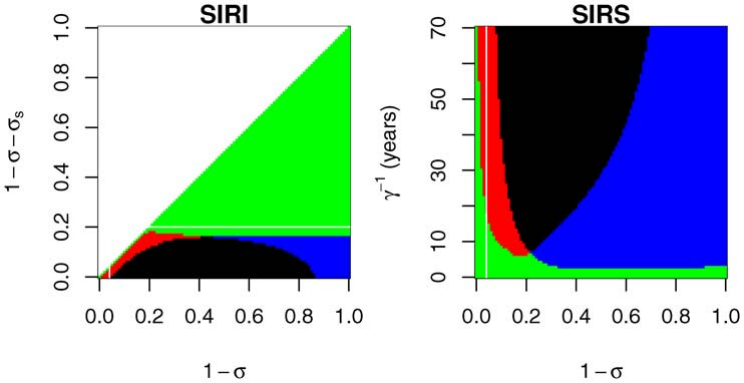
Effect of the introduction of within cluster gradual antigenic drift on the outcome of the invasion of a new antigenic cluster. Comparison of the 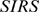 model (right) described in figure 7 and Supporting Information S1 with the 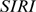 model of [28] (left). x-axis scale the amount of immune escape achieved by the mutant antigenic cluster. y-axis represent a measure of within cluster antigenic drift (see Supporting Information S1 for details). Colours: both antigenic clusters go extinct (black), the resident cluster only goes extinct (successful replacement, red); the mutant cluster only goes extinct (blue); no cluster goes extinct (coexistence, green). Extinction threshold is set at 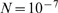. Parameter values are given in Table 1 (theoretical set). The horizontal white lines of the left graphs situates the reinfections thresholds of the 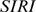 models (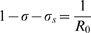). The vertical white lines set the highest immune escape intensity (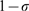) for which the same model without within cluster antigenic drift predicts replacements.

Contrary to the 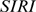 model which assumes that strains diversity within a given antigenic cluster results in partial immunity, the 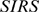 model considers that within antigenic clusters evolution results in a progressive loss of immunity [3]. Our investigation of the transient dynamics of drifting cross-reactive clusters modelled by 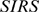 models as described in figure 7 and section 4 of Supporting Information S1 reveals that small amount of gradual antigenic drift can favour antigenic replacement over epidemic fadeout (figure 8 and Supporting Information S1). Within cluster gradual antigenic drift, whether included in 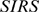 or 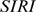 models can therefore turns epidemics fadeout of the mutant cluster into a successful replacement.

Introducing gradual antigenic drift in a minimal model for influenza also allows to reduce the high critical community size needed to ensure the persistence of a resident antigenic cluster. A small rate of gradual antigenic drift have a dramatic effect on the CCS of a resident antigenic cluster reducing the CCS from 10 millions to 1–2 millions (Supporting Information S1). CCS closer to one million renders stochastic effect (such as noise induced temporal asynchrony [23]) important to consider as they could potentially facilitate coexistence.

These theoretical results corroborate [52], [53] and [54] analysis of antigenic drift at the population level. [52] have estimated baseline antigenic drift rate from influenza like illness data using a model allowing sudden discrete changes and have shown that it was significantly different from zero. [53], using a method with a higher power of detection of positive selection than previous studies, have shown that within antigenic cluster change could be more important than traditionally (*e.g* [16]) believed.

Gradual antigenic drift should thus be part of a minimal model for influenza A along with punctuated immune escape.

#### Functional constraints

Functional constraints are well established for influenza A [19], [22], [45]. For instance, it has been established that cooperative activities of both HA and NA are critical for influenza virus infection and release [55]. Functional constraints can induce a fitness cost associated to an antigenic escape mutation. Lower fitness of the mutant cluster could be beneficial for the replacement dynamics as by decreasing the strength of the initial invasion, functional constraints could also decrease the risk of epidemic fadeout and long refractory periods that follow high depletion of susceptibles. A simple way to handle functional constraints is to consider a relation between the mutant cluster transmission rates (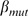) and its ability to escape previous immunity (governed by 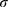). Without loss of generality, functional constraint can be introduced by lowering 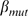 (assuming 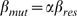 with 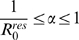) to ensure that 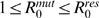. Using section Results results we can calculate the threshold value of 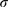, equal to 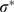, necessary for the antigenic cluster invasion. In case of both 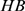 and 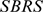 models, the threshold is defined by 
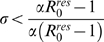
. Functional constraints can explain why immune escape mutations do not generate unrealistic high epidemics (Supporting Information S1). To compare our results to [14] model, we have neglected such constraint but they should be considered in further investigations. Such inclusion would need to incorporate compensatory mutations [19] to restore original function and re-increase the impaired 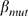.

#### Multiple infections before acquiring immunity

As we have shown through simulations (figures 2, 4 and 5), subtype replacement (as a consequence of antigenic shifts) appears impossible except in the case of the questionable 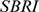 model. This is contrary to what have been observed during previous 1957's Asian flu and 1968's Hong Kong flu pandemics [56]. This lack of realism was reported by [7] in case of history based models and had been partially solved by including temporary cross-immunity [12]. However, other proposals that temporary cross-immunity could also be relevant. For instance, by using data of the first introduction of H3N2 type A influenza on the island of Tristan da Cunha in 1971, [57] show that two epidemics separated by 20 days only have affected the population and most of the hosts have been infected twice. This is different from the conventional knowledge of influenza immunology and suggests that multiple infection could be necessary before developing long term immunity. This creates far more susceptible individuals than expected from our models and greatly favours the persistence of the new subtype. It remains to be tested whether the persistence of the new subtype is sufficient to drive the resident subtype to extinction. Concerning epidemic influenza, the need to incorporate multiple infection before the acquisition of immunity deserves further attention.

As a last point, [58] have reopened a theory on influenza antigenic evolution dominant in 1960 [59]. Within this theory, the virus population is characterised by a limited set of antigenic types, all of which may be continuously (re-)generated from preexisting strains. [58] have shown that sampling from a population where a limited set of antigenic types describe complex dynamics can reproduce the specific patterns of antigenic cluster succession revealed by [6] analysis. This view offers an alternative explanation to the sequential antigenic drift scenario examined in this paper. Recent data, analysed by phylogenetic and coalescent based approaches, strongly suggest that influenza A dynamics is part of a source-sink system where the source could be a reservoir of a limited set of antigenic types [45], [60]–[65]. However, it remains to be seen to what extent restriction of viral genetic diversity could be achieved by [58] model. This model strongly depends on antigenic recycling to justify the low dimensionality of the phenotype space, but antigenic recycling does not seem to be supported by current data [11], [25].

In conclusion, our findings finally suggest the importance of gradual antigenic drift for epidemic dynamics even in the presence of punctuated immune escape. Our results indicate that status based model with reduced infectivity assumption can have profound consequences on the transient dynamics of strains invasion. In case of influenza, this model should be used with caution as it includes biologically unsupported processes that can induce serious bias.

## Supporting Information

Supporting Information S1 Influenza A gradual and epochal evolution: insights from simple models - 1. Reaction scheme for the SBRI model 2. Critical community size for influenza 3. Complementary results for the theoretical parameters set 4. A model for within cluster antigenic drift 5. Functional constraints
(3.19 MB PDF)
