# Supplementary material for: Influenza A Gradual and Epochal Evolution: Insights from Simple Models
Source: PLoS One. 2009 Oct 20;4(10):e7426. doi: 10.1371/journal.pone.0007426 (PMC2759541; doi:10.1371/journal.pone.0007426)
Supplement: Supporting Information S1 — Influenza A gradual and epochal evolution: insights from simple models - 1. Reaction scheme for the SBRI model 2. Critical community size for influenza 3. Complementary results for the theoretical parameters set 4. A model for within cluster antigenic drift 5. Functional constraints (3.19 MB PDF) [file pone.0007426.s001.pdf]

Supporting Information: Influenza A gradual and epochal evolution: insights from simple models

Sébastien Ballesteros[1,*], Elisabeta Vergu[2], Bernard Cazelles[1,3]

[1]UMR 7625 (UPMC, ENS, AgroParisTech, CNRS), Ecole Normale Supérieure, Unit of Eco-Evolutionary Mathematics, 46 rue d'Ulm, F-75230 Paris Cedex 05, France.
[2] INRA, UR341 Mathématiques et Informatique Appliquées, F-78352 Jouy en Josas, France
[3] UMMISCO UMI 209 IRD-UPMC, 93142 Bondy, France

[*] *Corresponding author*:
E-mail: sebastien.ballesteros@biologie.ens.fr

Contents

1 Reaction scheme for the *SBRI* model **2**

2 Critical community size for influenza **3**

3 Complementary results for the theoretical parameters set **4**

4 A model for within cluster antigenic drift **8**

5 Functional constraints **10**

1 Reaction scheme for the $SBRI$ model

reaction	rate
birth	
$R_\varnothing \to R_\varnothing + 1$	μN
death	
$R_\varnothing \to R_\varnothing - 1$	$\mu R_\varnothing$
$R_1 \to R_1 - 1$	μR_1
$R_1 \to R_2 - 1$	μR_2
$R_{12} \to R_{12} - 1$	μR_{12}
$I^1 \to I^1 - 1$	μI^1
$I^2 \to I^2 - 1$	μI^2
recovery	
$I^1 \to I^1 - 1$	νI^1
$I^2 \to I^2 - 1$	νI^2
I^1 production and R_1 only immunisation from $R_\varnothing$	
$R_\varnothing \to R_\varnothing - 1$; $R_1 \to R_1 + 1$; $I^1 \to I^1 + 1$	$(1-\sigma)\beta_1 R_\varnothing I^1/N$
I^2 production and R_1 only immunisation from $R_\varnothing$	
$R_\varnothing \to R_\varnothing - 1$; $R_1 \to R_1 + 1$; $I^2 \to I^2 + 1$	$(1-\sigma)\beta_2 R_\varnothing I^2/N$
I^1 production and R_{12} only immunisation from $R_\varnothing$	
$R_\varnothing \to R_\varnothing - 1$; $R_{12} \to R_{12} + 1$; $I^1 \to I^1 + 1$	$\sigma\beta_1 R_\varnothing I^1/N$
I^2 production and R_{12} only immunisation from $R_\varnothing$	
$R_\varnothing \to R_\varnothing - 1$; $R_{12} \to R_{12} + 1$; $I^2 \to I^2 + 1$	$\sigma\beta_2 R_\varnothing I^2/N$
I^1 production and R_{12} only immunisation from R_1	
$R_1 \to R_1 - 1$; $R_{12} \to R_{12} + 1$; $I^1 \to I^1 + 1$	$\beta_1 R_1 I^1/N$
I^2 production and R_{12} only immunisation from R_1	
$R_1 \to R_1 - 1$; $R_{12} \to R_{12} + 1$; $I^2 \to I^2 + 1$	$\beta_2 R_1 I^2/N$
R_{12} immunity acquisition from R_1 by I^1	
$R_{12} \to R_{12} + 1$; $R_1 \to R_1 - 1$	$\sigma\beta_1 R_1 I^1/N$
R_{12} immunity acquisition from R_1 by I^2	
$R_{12} \to R_{12} + 1$; $R_1 \to R_1 - 1$	$\sigma\beta_2 R_1 I^2/N$

Table S1: Reaction scheme for the $SBRI$ model

In the case of reduced susceptibility ($SBRS$ model), the reaction scheme of table S0 remains the same except that the last 2 reactions do not occur.

2 Critical community size for influenza

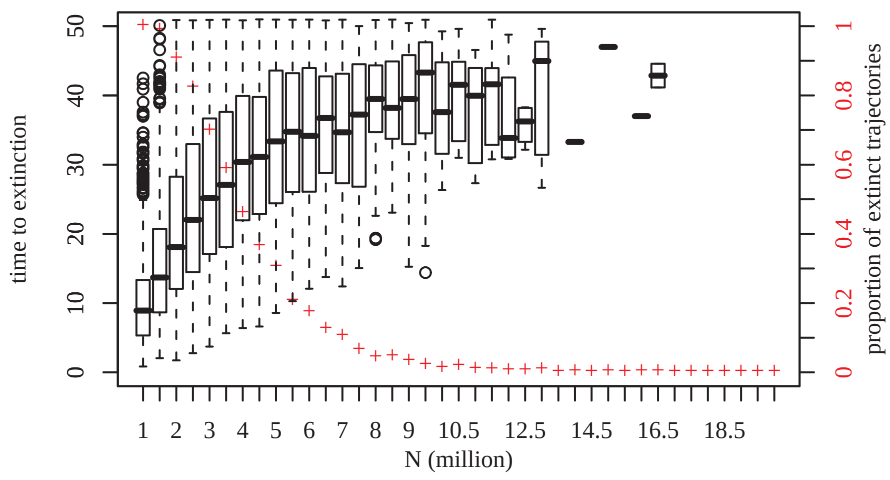

Figure S1: Endemic fadeouts obtained using the SIR stochastic model estimated by the distribution of the time to extinction. Red crosses represent the proportion of extinct trajectories after $T_{max} = 50$ years calculated on 1000 simulations. Initial conditions correspond to the endemic equilibrium of the deterministic model: $S = 200000$, $I = 250$ and $R = 799750$. Parameter values are given in Table 1 (theoretical set).

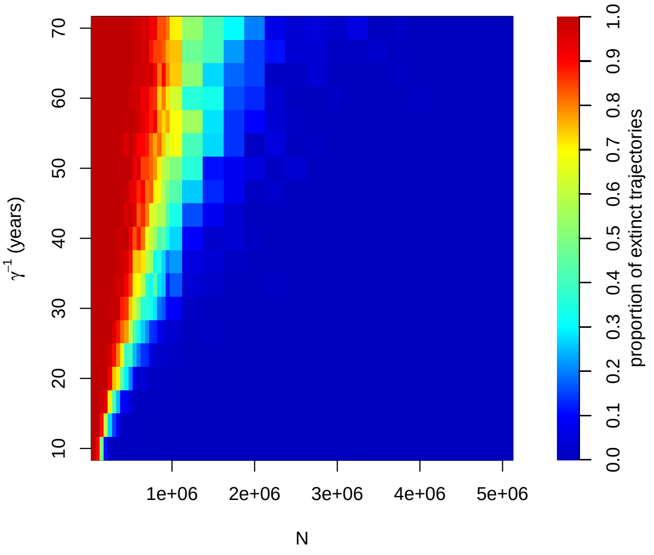

Figure S2: Effects of gradual antigenic drift on the CCS. We do not explicitly model mutant strains resulting from within gradual antigenic drift but consider that the emergence of new viral strains can be captured by introducing into the model a loss of immunity by the host, as originally suggested by Pease (1987). Gradual antigenic drift is therefore modelled by a $SIRS$ model with demography. Parameter γ governs the transition from R to S, reproducing gradual immune escape. The proportion of extinct trajectories after $T_{max} = 50$ years calculated on 100 simulations is plotted. Initial conditions correspond to the endemic equilibrium of the deterministic model. Parameter values are given in Table 1 (theoretical set).

3 Complementary results for the theoretical parameters set

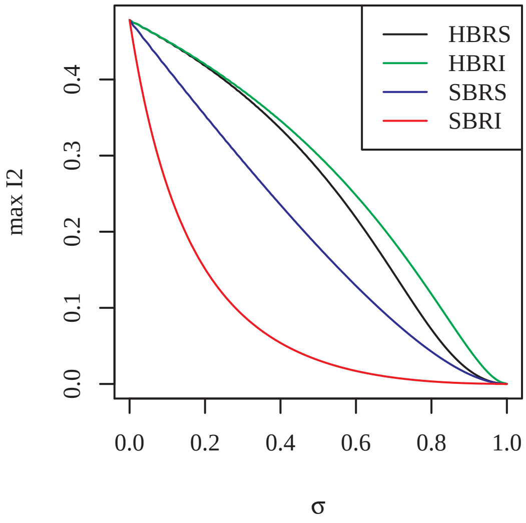

Figure S3: Peak value of the first outbreak of the mutant cluster as a function of partial cross-immunity (σ) for the four models studied. Parameter values are given in Table 1 (theoretical set). Initial conditions are : $I^1(0) = I^{1*} = 250.4 * 10^{-6}$, $I^2(0) = 10^{-6}$.

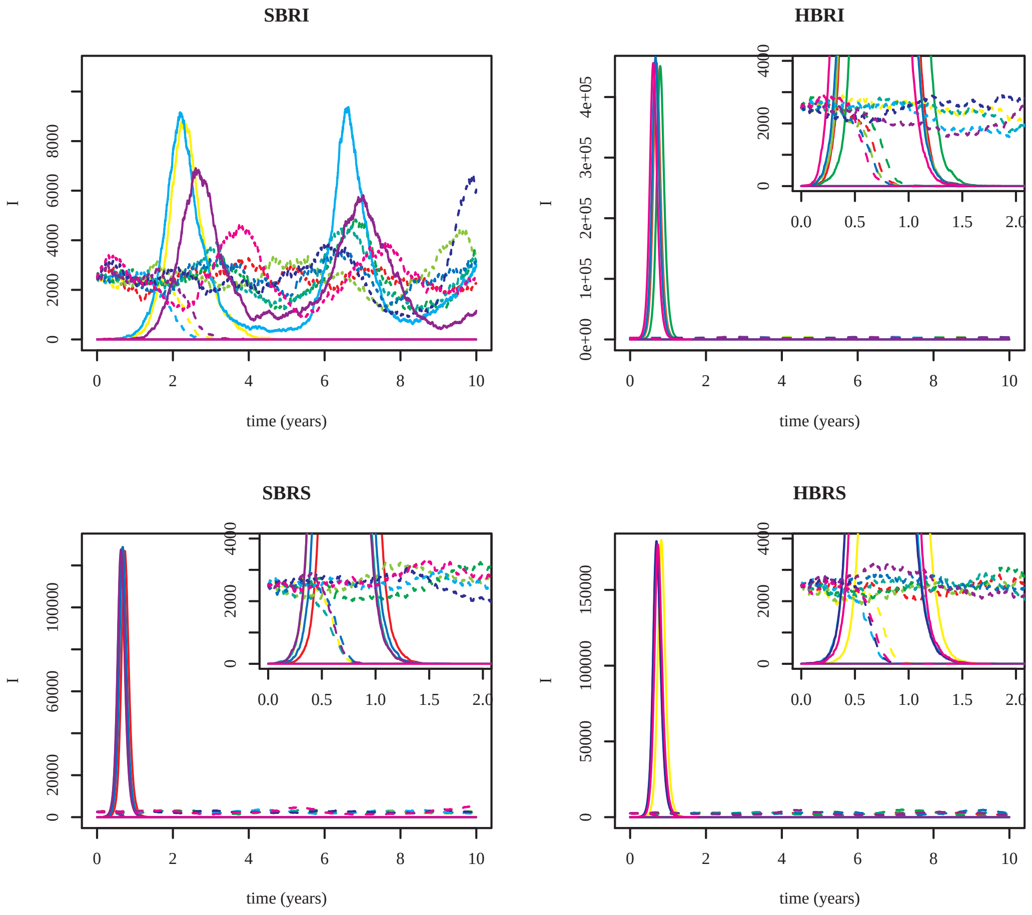

Figure S4: Ten realisations of the four stochastic models for $\sigma = 0.9$ (punctuated immune escape). Realisations are distinguished by different colours. Plain lines correspond to infectious hosts for the invader antigenic cluster and dashed lines to infectious hosts for the resident antigenic cluster.

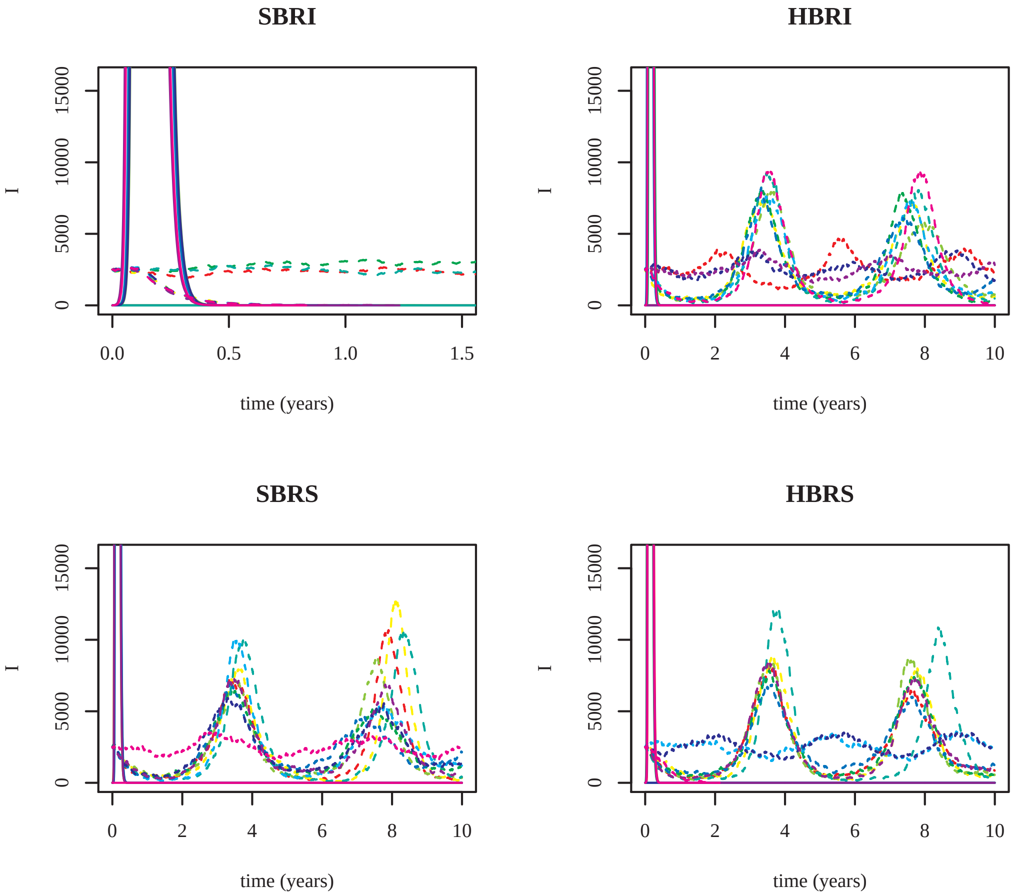

Figure S5: Ten realisations of the four stochastic models for $\sigma = 0.05$ (antigenic shift). Realisations are distinguished by different colours. Plain lines correspond to infectious hosts for the invader antigenic cluster and dashed lines to infectious hosts for the resident antigenic cluster.

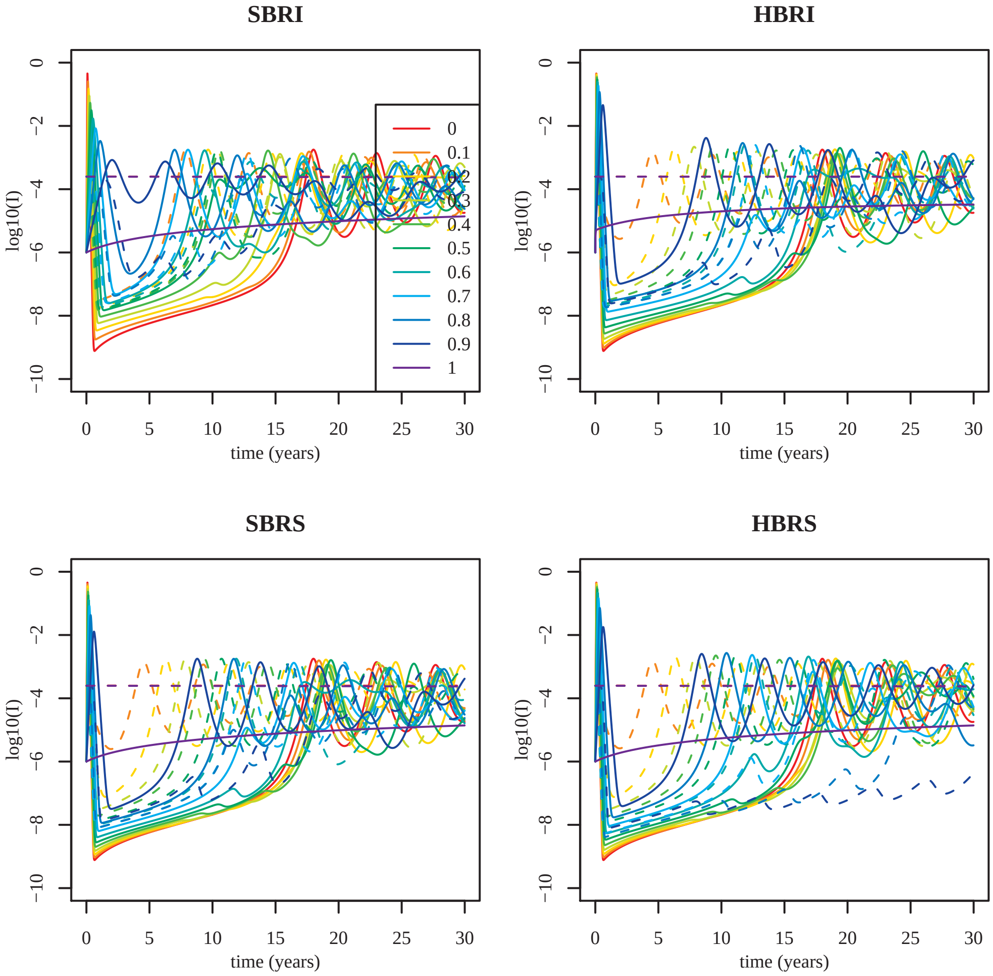

Figure S6: Transient invasion dynamics for the four two-cluster models studied in the presence of external reintroductions of infectious hosts. The decimal logarithm of the proportion of infectious hosts for the mutant antigenic cluster (plain lines) and for the resident cluster (dashed lines) is represented as a function of σ. Colours correspond to different partial cross-immunity (σ) values: from $\sigma = 0$ (antigenic shift, no cross-immunity) to $\sigma = 1$ (antigenic drift, full cross-immunity). Parameter values are given in Table 1 (theoretical set), $mp_i = 10^{-8}$. Initial conditions are : $I^1(0) = I^{1*} = 250.4 * 10^{-6}$, $I^2(0) = 10^{-6}$.

4 A model for within cluster antigenic drift

Within cluster antigenic drift is introduced by assuming that viruses present two parts:

- a conserved part, identical for both clusters whose phenotype cannot evolve.
- a specific part, subject to slight phenotypic variation following quasi-neutral mutation.

Partial cross-immunity is provided by the conserved part. Gradual antigenic drift induces continuous changes of the specific part and, new specific parts are introduced following immune escape mutations. We further assume that a primary immune response results in the acquisition of immune memory toward both the conserved and the specific parts.

We note σ and σ_s ($\sigma, \sigma_s \in [0,1]$) the degrees of protection provided respectively by immunity to the conserved and the specific part of the virus. We assume additivity of cross protection with the constraint that $\sigma + \sigma_s \leq 1$.

In case where we assume that within cluster antigenic drift results in strains diversity rendering intra-cluster immunity only partial, the previous assumption enables to recover Gökaydin *et al.* (2007) $SIRI$ model. Reinfections with strains belonging to a cluster for which hosts have been immunised occur with a reduced probability $(1 - (\sigma + \sigma_s))$. Infections by strains belonging to a mutant cluster never encountered by the hosts occur with a reduced probability $(1 - \sigma)$

In case where we assume that within antigenic clusters evolution results in a progressive loss of immunity as proposed by Pease (1987) ($SIRS$ framework) we introduce a parameter (γ) governing the rate of antigenic drift. Infections of naive hosts by strains belonging to cluster i confer full immunity toward strains of cluster i ($\sigma + \sigma_s = 1$, R_{Ci} hosts in figure 8 of the main text). Antigenic drift affects the specific part of the virus belonging to cluster i and induces a loss of immunity toward the specific part after a time governed by a rate γ ($R_{Ci} \rightarrow R_C$). R_C hosts can then be reinfected by strains belonging to cluster i but with a reduced probability $(1-\sigma)$ due to immunity provided by the conserved part. The conserved part also ensures that R_{Ci} and R_C hosts have a reduced probability $(1-\sigma)$ to be infected by strains belonging to cluster j (partial cross-immunity).

We formulate the model of figure 7 (main text) using an $HBRS$ framework and neglecting co-infections in order to compare it to Gökaydin *et al.* (2007) model. These assumptions lead to the following equations:

$$
\begin{aligned}
\dot{R_\varnothing} &= \mu - \beta_1 R_\varnothing (I^1_\varnothing + I^1_2) - \beta_2 R_\varnothing (I^2_\varnothing + I^2_1) - \mu R_\varnothing \\
\dot{I^1_\varnothing} &= \beta_1 R_\varnothing (I^1_\varnothing + I^1_2) + (1-\sigma)\beta_1 R_C (I^1_\varnothing + I^1_2) - \nu_1 I^1_\varnothing - \mu I^1_\varnothing \\
\dot{I^1_\varnothing} &= \beta_2 R_\varnothing (I^2_\varnothing + I^2_1) + (1-\sigma)\beta_2 R_C (I^2_\varnothing + I^2_1) - \nu_2 I^2_\varnothing - \mu I^2_\varnothing \\
\dot{R_{C1}} &= \nu_1 I^1_\varnothing - (1-\sigma)\beta_2 R_{C1} (I^2_\varnothing + I^2_1) - \gamma_1 R_{C1} + \gamma_2 R_{C12} - \mu R_{C1} \\
\dot{R_{C2}} &= \nu_2 I^2_\varnothing - (1-\sigma)\beta_1 R_{C2} (I^1_\varnothing + I^1_2) - \gamma_2 R_{C2} + \gamma_1 R_{C12} - \mu R_{C2} \\
\dot{R_C} &= \gamma_1 R_{C1} + \gamma_2 R_{C2} - (1-\sigma)\beta_1 R_C (I^1_\varnothing + I^1_2) - (1-\sigma)\beta_2 R_C (I^2_\varnothing + I^2_1) - \mu R_C \\
\dot{I^1_2} &= (1-\sigma)\beta_1 R_{C2} (I^1_\varnothing + I^1_2) - \nu_1 I^1_2 - \mu I^1_2 \\
\dot{I^2_1} &= (1-\sigma)\beta_2 R_{C1} (I^2_\varnothing + I^2_1) - \nu_2 I^2_1 - \mu I^2_1 \\
\dot{R_{C12}} &= \nu_1 I^1_2 + \nu_2 I^2_1 - \gamma_2 R_{C12} - \gamma_1 R_{C12} - \mu R_{C12}
\end{aligned}
$$

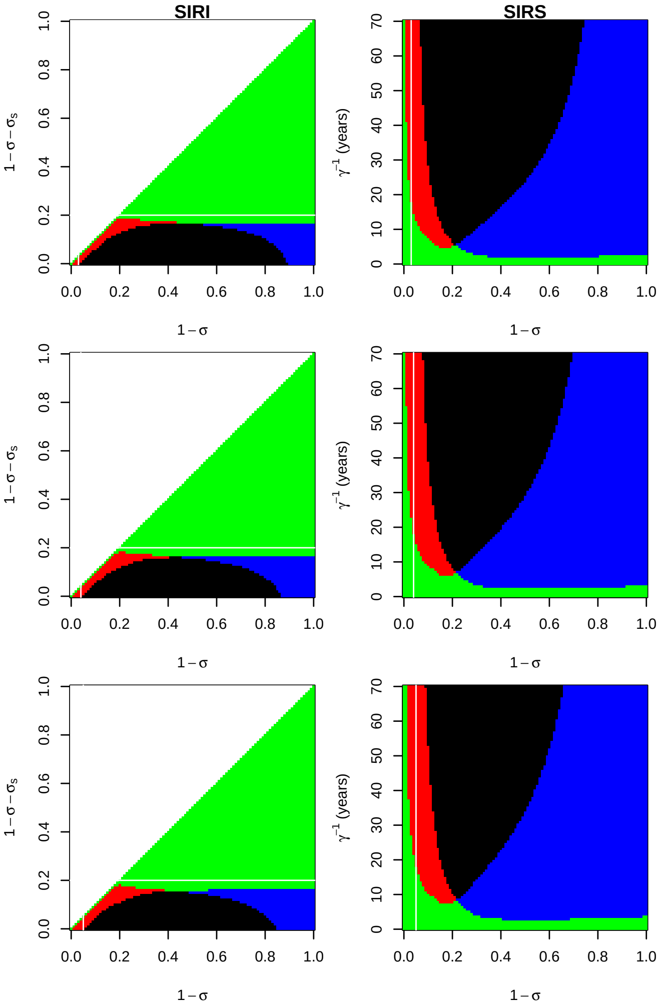

Figure S7: Effect of the introduction of within cluster gradual antigenic drift on the outcome of the invasion of a new antigenic cluster. Comparison of the $SIRS$ model (right) described in figure 7 and section 4 of the appendix with the $SIRI$ model of Gökaydin *et al.* (2007) (left). x-axis scale the amount of immune escape achieved by the mutant antigenic cluster. y-axis represent a measure of within cluster antigenic drift (see section 3 of the appendix for details). Colours: both antigenic clusters go extinct (black), the resident cluster only goes extinct (successful replacement, red); the mutant cluster only goes extinct (blue); no cluster goes extinct (coexistence, green). Extinction threshold is set at $N = 10^{-6}$ (top panels), $N = 10^{-7}$ (middle panels) and $N = 10^{-8}$ (bottom panels). Parameter values are given in Table 1 (theoretical set). The horizontal white lines of the right graphs situates the reinfections thresholds of the $SIRI$ models $(1 - \sigma - \sigma_s = \frac{1}{R_0})$. The vertical white lines set the highest immune escape intensity $(1 - \sigma)$ for which the same model without within cluster antigenic drift predicts replacements.

5 Functional constraints

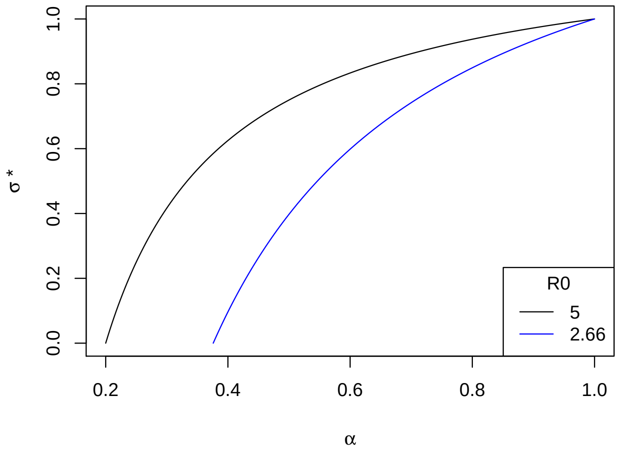

Figure S8: Effect of the fitness reduction (mutant transmission rate β reduced by a factor α) associated with immune escape plotted for different values of the resident cluster R_0. The threshold value of σ (σ^*) needed to ensure the mutant cluster invasion is plotted against α.
